# Supplementary material for: MetaRibo-Seq measures translation in microbiomes
Source: Nat Commun. 2020 Jun 29;11:3268. doi: 10.1038/s41467-020-17081-z (PMC7324362; doi:10.1038/s41467-020-17081-z)
Supplement: Supplementary file 10 — Supplementary Data 7 [file 41467_2020_17081_MOESM10_ESM.zip › File2/Confidence_High_Taxonomy/421067_out.krona.html]

Javascript must be enabled to view this page.

members
magnitude
magnitudeUnassigned
count
unassigned
taxon
rank

421067\_out

271

superkingdom
271
2

976
271
phylum

class
200643
271

order
271
171549

28
815
family

genus
28
816

species

SRS144537\_contig\_number\_28003SRS149325\_contig\_number\_12141
2
291644

species

SRS148817\_contig\_number\_9739
246787
1

1
310297

SRS023583\_contig\_number\_5910
species

46506
1

SRS144506\_contig\_number\_15218
species

1638780
15

SRS013098\_contig\_number\_29905SRS016753\_contig\_number\_9308SRS017859\_contig\_number\_4885SRS018049\_contig\_number\_115SRS045739\_contig\_number\_10459SRS051882\_contig\_number\_10735SRS052078\_contig\_number\_2628SRS057480\_contig\_number\_75SRS063190\_contig\_number\_contig-100\_189.97171SRS063985\_contig\_number\_29378SRS074670\_contig\_number\_4323SRS101268\_contig\_number\_contig-100\_998.999SRS1041116\_contig\_number\_3384SRS143466\_contig\_number\_3079SRS148159\_contig\_number\_42121
species

species

SRS019808\_contig\_number\_13944SRS062654\_contig\_number\_20932SRS103987\_contig\_number\_24547SRS146764\_contig\_number\_7592
821
4

species

SRS050941\_contig\_number\_6515SRS055966\_contig\_number\_1235SRS140492\_contig\_number\_22733
3
817


SRS143991\_contig\_number\_17351
species
1739517
1

family
3
171552

genus
3
838

species

SRS023914\_contig\_number\_24341SRS049995\_contig\_number\_24669SRS053398\_contig\_number\_3624
3
165179

171551
1
family


SRS055017\_contig\_number\_16840
species
1
2049046

family
2005525
239

genus
239
375288

SRS014923\_contig\_number\_29900
1

387661
14

SRS015960\_contig\_number\_17628SRS016954\_contig\_number\_26495SRS019693\_contig\_number\_6942SRS021484\_contig\_number\_1676SRS021948\_contig\_number\_6390SRS023715\_contig\_number\_7260SRS043768\_contig\_number\_contig-100\_861.186177SRS056259\_contig\_number\_14751SRS075878\_contig\_number\_3038SRS077127\_contig\_number\_10957SRS104084\_contig\_number\_13191SRS1041129\_contig\_number\_10962SRS1041145\_contig\_number\_18599SRS893342\_contig\_number\_1006
species


SRS011302\_contig\_number\_6666SRS011405\_contig\_number\_16491SRS012969\_contig\_number\_22177SRS013476\_contig\_number\_41633SRS013521\_contig\_number\_11383SRS013687\_contig\_number\_30594SRS013800\_contig\_number\_13406SRS013940\_contig\_number\_20971SRS013951\_contig\_number\_28531SRS014235\_contig\_number\_25610SRS014287\_contig\_number\_16556SRS014412\_contig\_number\_796SRS014459\_contig\_number\_17495SRS014613\_contig\_number\_17622SRS014683\_contig\_number\_14892SRS014948\_contig\_number\_16147SRS015065\_contig\_number\_21242SRS015264\_contig\_number\_18645SRS015431\_contig\_number\_57887SRS015579\_contig\_number\_12533SRS015663\_contig\_number\_14086SRS015694\_contig\_number\_8071SRS015782\_contig\_number\_14087SRS016018\_contig\_number\_contig-100\_1037.100105SRS016056\_contig\_number\_3747SRS016132\_contig\_number\_4219SRS016203\_contig\_number\_11470SRS016335\_contig\_number\_28616SRS016381\_contig\_number\_2543SRS016840\_contig\_number\_contig-100\_357.28796SRS017103\_contig\_number\_6178SRS017247\_contig\_number\_8260SRS017433\_contig\_number\_6163SRS017521\_contig\_number\_30989SRS017701\_contig\_number\_9809SRS017821\_contig\_number\_23357SRS018133\_contig\_number\_7204SRS018575\_contig\_number\_5952SRS018836\_contig\_number\_20186SRS018888\_contig\_number\_7651SRS018936\_contig\_number\_8858SRS019030\_contig\_number\_14592SRS019068\_contig\_number\_73511SRS019161\_contig\_number\_18761SRS019178\_contig\_number\_2213SRS019286\_contig\_number\_5216SRS019381\_contig\_number\_14090SRS019397\_contig\_number\_9568SRS019445\_contig\_number\_27699SRS019910\_contig\_number\_23176SRS019968\_contig\_number\_16895SRS020233\_contig\_number\_9864SRS020271\_contig\_number\_132SRS020328\_contig\_number\_22154SRS022137\_contig\_number\_2336SRS022524\_contig\_number\_14185SRS023526\_contig\_number\_24081SRS024009\_contig\_number\_2822SRS024075\_contig\_number\_14050SRS024132\_contig\_number\_41702SRS024331\_contig\_number\_34808SRS024435\_contig\_number\_6795SRS024492\_contig\_number\_16684SRS042966\_contig\_number\_18859SRS043667\_contig\_number\_contig-100\_905.42339SRS043841\_contig\_number\_117SRS044535\_contig\_number\_3479SRS045004\_contig\_number\_29910SRS045244\_contig\_number\_6990SRS045645\_contig\_number\_18613SRS045713\_contig\_number\_12381SRS045826\_contig\_number\_2463SRS046068\_contig\_number\_974SRS046369\_contig\_number\_9140SRS046717\_contig\_number\_7491SRS047014\_contig\_number\_31564SRS047044\_contig\_number\_5610SRS047433\_contig\_number\_13075SRS048164\_contig\_number\_15440SRS048870\_contig\_number\_20264SRS048981\_contig\_number\_6554SRS049402\_contig\_number\_9038SRS049446\_contig\_number\_10923SRS049712\_contig\_number\_17670SRS049773\_contig\_number\_20304SRS049896\_contig\_number\_21383SRS049959\_contig\_number\_47813SRS050026\_contig\_number\_708SRS050299\_contig\_number\_14506SRS050422\_contig\_number\_33859SRS050520\_contig\_number\_contig-100\_542.21927SRS050752\_contig\_number\_27665SRS050925\_contig\_number\_17934SRS051031\_contig\_number\_23682SRS051610\_contig\_number\_5544SRS052697\_contig\_number\_26708SRS053356\_contig\_number\_11484SRS053649\_contig\_number\_contig-100\_1605.142834SRS054059\_contig\_number\_4390SRS054352\_contig\_number\_contig-100\_897.120243SRS054905\_contig\_number\_17771SRS055982\_contig\_number\_194SRS056519\_contig\_number\_17403SRS058070\_contig\_number\_20825SRS058770\_contig\_number\_16609SRS062427\_contig\_number\_9450SRS063040\_contig\_number\_29564SRS063127\_contig\_number\_2819SRS063489\_contig\_number\_2010SRS064276\_contig\_number\_12337SRS064557\_contig\_number\_18238SRS064757\_contig\_number\_8824SRS065176\_contig\_number\_14795SRS065397\_contig\_number\_5026SRS065504\_contig\_number\_10645SRS075021\_contig\_number\_4507SRS075341\_contig\_number\_22164SRS075773\_contig\_number\_contig-100\_695.226114SRS075821\_contig\_number\_22312SRS075984\_contig\_number\_2659SRS076804\_contig\_number\_16008SRS076876\_contig\_number\_536SRS076976\_contig\_number\_17662SRS077024\_contig\_number\_3527SRS077086\_contig\_number\_11958SRS077231\_contig\_number\_23189SRS077552\_contig\_number\_27283SRS077589\_contig\_number\_2168SRS077641\_contig\_number\_21903SRS077849\_contig\_number\_24796SRS078176\_contig\_number\_13980SRS078242\_contig\_number\_11882SRS078419\_contig\_number\_22453SRS078665\_contig\_number\_7624SRS097920\_contig\_number\_contig-100\_637.189880SRS097958\_contig\_number\_13367SRS098061\_contig\_number\_10244SRS098073\_contig\_number\_16871SRS098571\_contig\_number\_65225SRS098644\_contig\_number\_39952SRS098655\_contig\_number\_11888SRS098717\_contig\_number\_12510SRS101376\_contig\_number\_15383SRS101433\_contig\_number\_8713SRS104036\_contig\_number\_4192SRS1041031\_contig\_number\_14037SRS1041039\_contig\_number\_11360SRS1041091\_contig\_number\_14095SRS1041092\_contig\_number\_137SRS1041112\_contig\_number\_5392SRS1041118\_contig\_number\_3250SRS1041130\_contig\_number\_4876SRS1041134\_contig\_number\_1689SRS1041136\_contig\_number\_15699SRS1041137\_contig\_number\_14131SRS1041138\_contig\_number\_4632SRS1041139\_contig\_number\_1138SRS1041140\_contig\_number\_21466SRS1041141\_contig\_number\_8170SRS104311\_contig\_number\_18634SRS104327\_contig\_number\_contig-100\_570.47278SRS104400\_contig\_number\_23035SRS104636\_contig\_number\_24111SRS104912\_contig\_number\_17212SRS104975\_contig\_number\_17858SRS105082\_contig\_number\_9066SRS105153\_contig\_number\_31765SRS1054716\_contig\_number\_15984SRS1055022\_contig\_number\_14765SRS1055069\_contig\_number\_12501SRS1055076\_contig\_number\_11293SRS1055095\_contig\_number\_317SRS140513\_contig\_number\_12844SRS142503\_contig\_number\_28492SRS142542\_contig\_number\_24997SRS142890\_contig\_number\_24727SRS142923\_contig\_number\_21838SRS143070\_contig\_number\_contig-100\_655.110476SRS143148\_contig\_number\_22814SRS143181\_contig\_number\_1921SRS143372\_contig\_number\_4992SRS143523\_contig\_number\_15777SRS143722\_contig\_number\_15492SRS143780\_contig\_number\_22178SRS143876\_contig\_number\_14657SRS143895\_contig\_number\_46661SRS144135\_contig\_number\_27256SRS144297\_contig\_number\_8850SRS144603\_contig\_number\_2378SRS144714\_contig\_number\_29172SRS145308\_contig\_number\_4114SRS145497\_contig\_number\_18900SRS146812\_contig\_number\_contig-100\_786.316506SRS146813\_contig\_number\_contig-100\_1774.142591SRS147139\_contig\_number\_27694SRS147271\_contig\_number\_40021SRS147346\_contig\_number\_60615SRS147425\_contig\_number\_8911SRS147557\_contig\_number\_29902SRS147614\_contig\_number\_7328SRS147653\_contig\_number\_contig-100\_965.34585SRS147977\_contig\_number\_11759SRS148091\_contig\_number\_10064SRS148196\_contig\_number\_35176SRS148253\_contig\_number\_12645SRS148319\_contig\_number\_11782SRS148424\_contig\_number\_18366SRS148721\_contig\_number\_46164SRS148784\_contig\_number\_24312SRS149075\_contig\_number\_17159SRS149181\_contig\_number\_25182SRS893170\_contig\_number\_10880SRS893230\_contig\_number\_2172SRS893252\_contig\_number\_13884SRS893292\_contig\_number\_6608SRS893300\_contig\_number\_2321SRS893327\_contig\_number\_4987SRS893358\_contig\_number\_6763SRS893378\_contig\_number\_13169SRS893382\_contig\_number\_5612SRS893383\_contig\_number\_36614
species
46503
221

3
1750560
species

SRS012849\_contig\_number\_30963SRS012849\_contig\_number\_contig-100\_746.200943SRS893366\_contig\_number\_18082
